# Supplementary material for: Enzymatic saccharification of peat polysaccharides is limited by accessibility
Source: PLoS One. 2025 May 23;20(5):e0312219. doi: 10.1371/journal.pone.0312219 (PMC12101845; doi:10.1371/journal.pone.0312219)
Supplement: S1 Fig — (PDF) [file pone.0312219.s001.pdf]

A)

Pretreated wheat straw

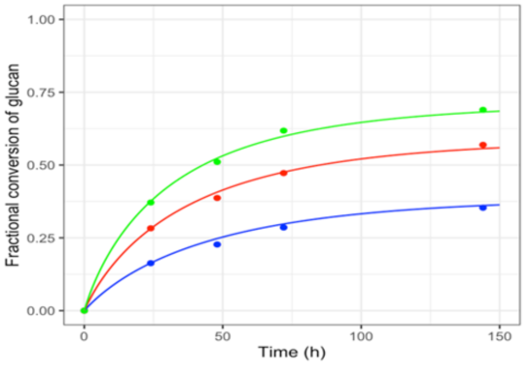

k_i (x 10^{-3} h^{-1}): 17.6 ± 5.25

k_s (h^{-1}): 15.033 ± 2.67

B)

peat pretreated at 180 °C

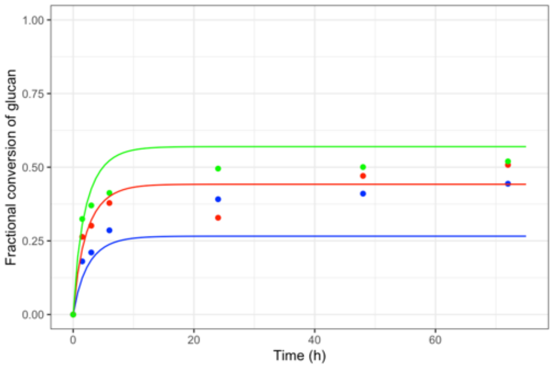

k_i (x 10^{-3} h^{-1}): 351.71 ± 82.2

k_s (h^{-1}): 118.95 ± 25.10

S1 Fig. Estimation of the inactivation (k_i) and the catalytic (k_s) rate constants. To estimate k_i and k_s (presented with ± SE), a two-stage kinetic model for synergistic release of glucose was applied on fractional conversion data from A) peat pretreated at 180 °C and B) pretreated wheat straw saccharified over 144 hours with 5 (blue), 10 (red) and 15 (green) mg CTec3 / g DM. The model fit for wheat straw serves as validation of the model. The model is not fit to estimate k_i and k_s values for peat pretreated at 180 °C due to the poor curve fit.
