# Supplementary material for: Enzymatic saccharification of peat polysaccharides is limited by accessibility
Source: PLoS One. 2025 May 23;20(5):e0312219. doi: 10.1371/journal.pone.0312219 (PMC12101845; doi:10.1371/journal.pone.0312219)
Supplement: S2 Fig — (PDF) [file pone.0312219.s002.pdf]

Average	mg/kg	mg/kg	mg/kg	mg/kg	mg/kg	mg/kg	mg/kg	mg/kg	mg/kg	mg/kg	mg/kg	mg/kg
	Borum	Sodíum	Magnesium	Aluminium	Phosphorus	Sulphur	Potassium	Calcium	Vanadium	Chromium	Manganese	Iron
Catotelm	20.6	131.5	1911.1	2751.5	565.4	4772.3	589.3	26906.4	17.0	3.5	42.4	4026.8
Peat	3.9	145.8	1181.4	1773.7	517.8	1878.2	1066.3	6719.7	4.0	3.4	65.4	1956.2
peat pretreated at 180 °C	3.9	179.8	1146.8	1726.6	477.9	1964.3	1039.6	6634.3	4.1	9.7	65.0	2057.9
peat pretreated at 180 °C and washed	3.0	12.2	225.8	952.6	67.4	1196.6	176.8	2793.0	2.6	2.8	26.3	1108.3
Pretreated wheat straw	5.4	25.4	149.5	260.9	168.8	645.3	722.0	1903.6	0.4	3.3	8.9	638.6
	mg/kg	mg/kg	mg/kg	mg/kg	mg/kg	mg/kg	mg/kg	mg/kg	mg/kg	mg/kg	mg/kg	mg/kg
	Cobalt	Nikkel	Copper	Zinc	Arsenic	Selenium	Strontium	Molybdenum	Cadmium	Barium	Thallium	Lead
Catotelm	1.0	9.1	39.1	19.7	1.9	1.7	129.8	1.2	0.2	62.5	0.0	3.1
Peat	0.6	2.1	7.5	32.8	1.2	0.7	28.1	1.3	0.2	12.6	0.0	10.8
peat pretreated at 180 °C	0.7	9.3	8.3	41.2	1.3	0.6	28.9	2.5	0.2	12.0	0.0	11.9
peat pretreated at 180 °C and washed	0.3	4.3	5.5	20.8	0.4	0.3	13.2	1.1	0.1	2.0	0.0	7.3
Pretreated wheat straw	0.1	1.4	4.2	29.5	0.1	0.0	8.9	0.4	0.1	26.0	0.0	0.3

Standard deviation	mg/kg	mg/kg	mg/kg	mg/kg	mg/kg	mg/kg	mg/kg	mg/kg	mg/kg	mg/kg	mg/kg	mg/kg
	Borum	Sodíum	Magnesium	Aluminium	Phosphorus	Sulphur	Potassium	Calcium	Vanadium	Chromium	Manganese	Iron
Catotelm	0.6	0.5	23.8	59.1	5.1	69.8	15.7	23.5	0.4	0.2	1.4	78.7
Peat	0.2	7.9	73.8	140.7	17.0	25.0	47.1	499.3	0.2	0.1	7.3	158.7
peat pretreated at 180 °C	0.4	39.1	37.3	23.8	19.0	143.1	19.4	169.1	0.1	0.5	1.0	148.2
peat pretreated at 180 °C and washed	0.3	8.4	12.1	59.1	2.6	107.2	23.3	107.5	0.0	0.3	1.4	46.7
Pretreated wheat straw	9.1	13.9	145.4	352.5	1.8	44.9	26.7	852.9	0.1	1.8	0.7	6.4
	mg/kg	mg/kg	mg/kg	mg/kg	mg/kg	mg/kg	mg/kg	mg/kg	mg/kg	mg/kg	mg/kg	mg/kg
	Cobalt	Nikkel	Copper	Zinc	Arsenic	Selenium	Strontium	Molybdenum	Cadmium	Barium	Thallium	Lead
Catotelm	0.0	0.2	0.2	0.4	0.0	0.0	1.1	0.0	0.0	1.2	0.0	0.0
Peat	0.0	0.1	0.3	0.6	0.0	0.0	0.8	0.1	0.0	2.1	0.0	0.4
peat pretreated at 180 °C	0.0	0.2	0.2	11.3	0.1	0.0	0.8	0.0	0.0	1.2	0.0	0.8
peat pretreated at 180 °C and washed	0.0	0.7	0.6	0.8	0.0	0.0	0.6	0.1	0.0	0.6	0.0	0.3
Pretreated wheat straw	0.0	0.3	0.0	34.4	0.0	0.0	4.9	0.0	0.0	0.2	0.0	0.1

S2 Fig. Metal concentration (mg/Kg) in substrates determined with inductively coupled plasma-mass spectrometry (ICP-MS). Prior to analysis the substrates were freeze-dried and ground with a mortar and pestle. 0.3 g sample was weighted into a teflonbomb and added 10 ml of concentrated nitric acid. The samples was digested in a microwave with a ramping phase of 20 minutes and a hold-phase of 25 minutes at 180 °C. After cooling, the solutions were first diluted to 50 ml with MilliQ water and further diluted times 10 before analysis. The values presented are averages of three replicates ± standard deviation.
