# Supplementary material for: Enzymatic saccharification of peat polysaccharides is limited by accessibility
Source: PLoS One. 2025 May 23;20(5):e0312219. doi: 10.1371/journal.pone.0312219 (PMC12101845; doi:10.1371/journal.pone.0312219)
Supplement: S3 Fig — (PDF) [file pone.0312219.s003.pdf]

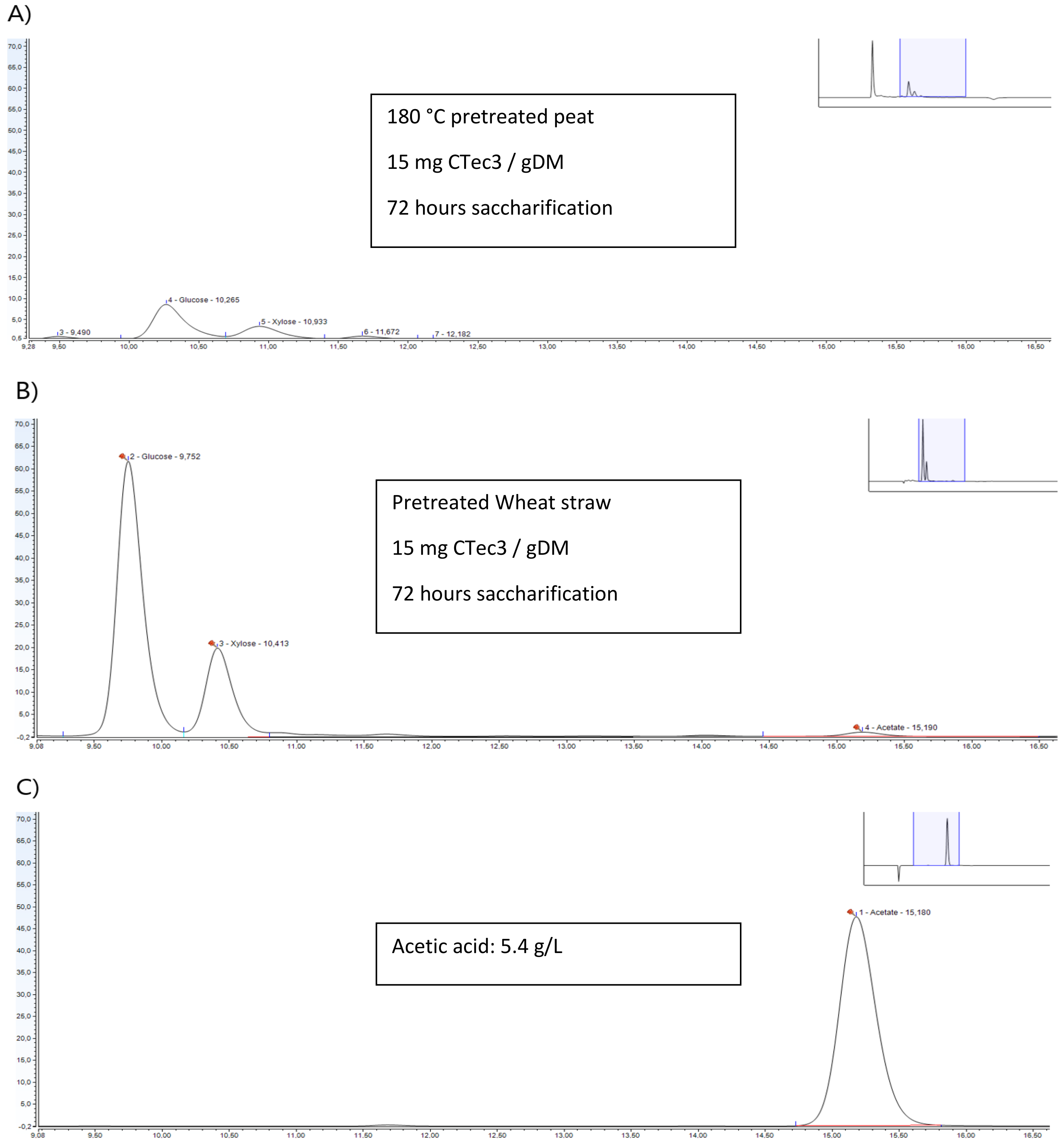

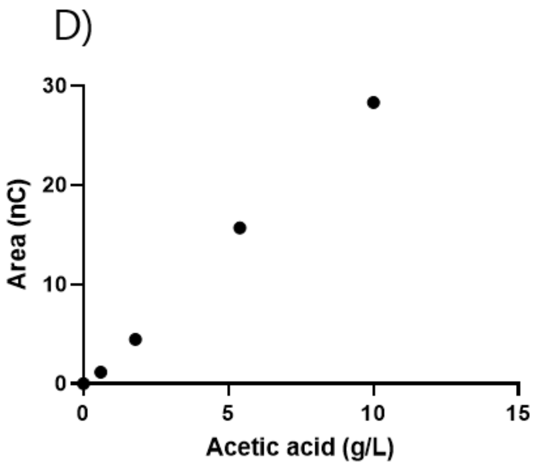

S3 Fig. Detection of acetic acid. Samples of peat pretreated at 180 °C and pretreated wheat straw taken after 72 hours of incubation with CTec3 were analyzed for acetic acid using a Dionex Ultimate 3000 system (Thermo Scientific) as described in the methods section. Chromatograms were obtained from analyzing A) 180 °C pretreated peat and B) pretreated wheat straw that were diluted the same and C) an acetic acid solution of 5.4 g/L prepared in MilliQ water. D) Acetic acid standard curve with concentrations of 0, 0.6, 1.8, 5.4 and 10 g/L.
