# Supplementary material for: Enzymatic saccharification of peat polysaccharides is limited by accessibility
Source: PLoS One. 2025 May 23;20(5):e0312219. doi: 10.1371/journal.pone.0312219 (PMC12101845; doi:10.1371/journal.pone.0312219)
Supplement: S4 Fig — (PDF) [file pone.0312219.s004.pdf]

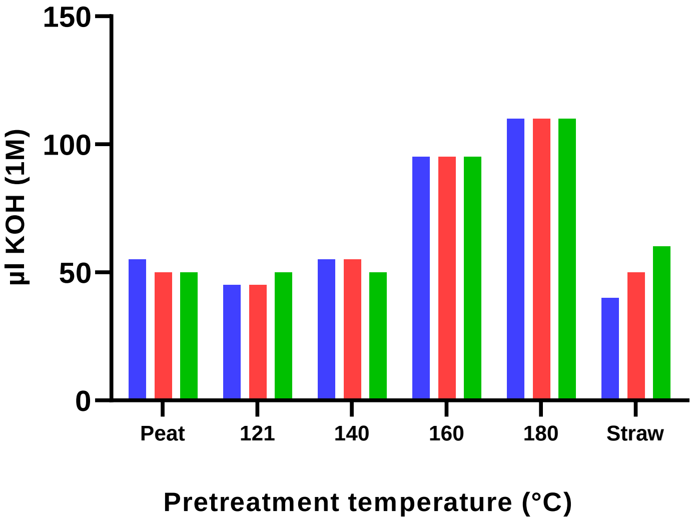

S4 Fig. Effect of pretreatment temperature on titrant requirements during saccharification. Duplicate samples of untreated peat, 121 °C, 140 °C, 160 °C and 180 °C pretreated peat and pretreated wheat straw was incubated for 24 hours with 5 (blue), 10 (red) and 15 (green) mg CTec3 / g DM for 24 hours, 50 ˚C, pH 5.2. During incubation, pH was adjusted to 5.2 with KOH (1M) after 1.5, 6 and 24 hours. The same amount of KOH (1M) was added to both duplicate samples.
