# Supplementary material for: Enzymatic saccharification of peat polysaccharides is limited by accessibility
Source: PLoS One. 2025 May 23;20(5):e0312219. doi: 10.1371/journal.pone.0312219 (PMC12101845; doi:10.1371/journal.pone.0312219)
Supplement: S5 Fig — (PDF) [file pone.0312219.s005.pdf]

A)

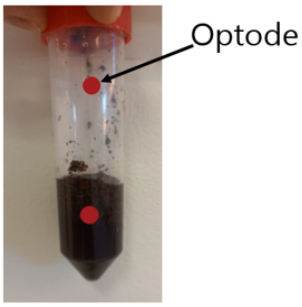

B)

O_2 saturation (%)	Air %		N2%	
Time	Slurry	Headspace	Slurry	Headspace
0 min (just before incubation)	2,0	77,6	5,9	5,5
5 min	85,6	88,1	2,2	5,3
60 min	13,4	13,1	0,73	0,57
120 min	13,6	13,6	0,73	0,84

C)

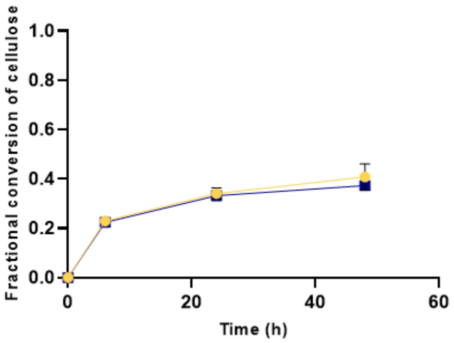

D)

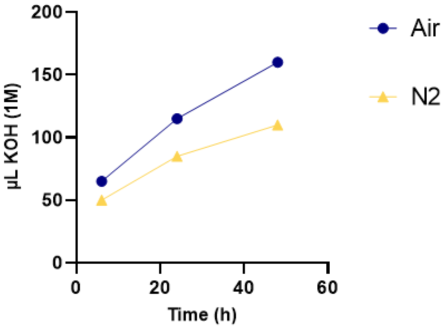

S5 Fig. Effect of dissolved oxygen on saccharafication and KOH (1M) requirements of peat pretreated at 180 °C. Dissolved oxygen measurements are presented as oxygen saturation (%) compared to air, where the saturation in air is 100%. A Fibox 4 oxygen meter (Presense, Germany) and optodes glued on the inside of the experimental tubes was used to optically measure oxygen saturation (%). A) Placement of oxygen optodes in experimental tubes. B) Oxygen saturation in slurry and headspace of experimental tubes after 5, 60 and 120 minutes incubation in a hybridization oven at 50 °C, pH 5.2. The experimental tubes were mixed horizontally at 40 rpm. C) Fractional conversion of peat pretreated at 180 °C incubated for 48 hours with 5 mg CTec3 / DM at ambient air in headspace (blue) and with N2-flushed headspace (yellow) at 50 ˚C, pH 5.2. D) KOH (1M) requirements for keeping pH at 5.2 during the 48 hours of incubation with ambient air in headspace (blue) and with nitrogen flushed headspace (yellow).
