# Supplementary material for: Enzymatic saccharification of peat polysaccharides is limited by accessibility
Source: PLoS One. 2025 May 23;20(5):e0312219. doi: 10.1371/journal.pone.0312219 (PMC12101845; doi:10.1371/journal.pone.0312219)
Supplement: S6 Fig — (PDF) [file pone.0312219.s006.pdf]

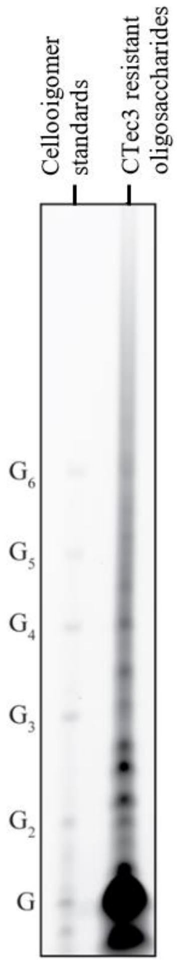

S6 Fig. CTec3 resistant oligosaccharides from saccharification of 180 °C pretreated peat analysed by polysaccharide analysis with carbohydrate gel electrophoresis (PACE). A range of oligosaccharides that do not co-migrate with cello-oligosaccharide standards are released by CTec3 enzyme cocktail. Cello-oligosaccharides: G-G_6.
