# Supplementary material for: Enzymatic saccharification of peat polysaccharides is limited by accessibility
Source: PLoS One. 2025 May 23;20(5):e0312219. doi: 10.1371/journal.pone.0312219 (PMC12101845; doi:10.1371/journal.pone.0312219)

Supporting information: R material

Hydrothermal pretreatment renders peat susceptible to enzymatic saccharification by J. Thomsen, S. Lett, H. Martens, H. Sørensen, D. Kelleher, T. Tryfona, P. Dupree, K.S.Johansen

Helle Sørensen, Data Science Lab, University of Copenhagen

July 2024

Contents

About this document . . . 1
Load packages . . . 1
Load and plot data . . . 1
External/fixed parameters . . . 3
ODE . . . 4
Non-linear regression . . . 5
Estimation for our data . . . 7
Plot of solution . . . 7

About this document

This document shows and explains the R code used to (a) solve the two-stage kinetic model for fixed parameters values and (b) estimate the parameters *k_i* and *k_s* (called *kia* and *ks* in the code below). See Section 2.11 in the main text and Fig SI.1 in the Supplement.

The code combines an ODE solver with non-linear regression. Unfortunately the ODE solver and the standard function for non-linear regression (*nls*) apparently do not work well together, so I have made a manual implementation of the regression part. This makes the code more difficult to read compared to a situation where *nls* could have been applied.

Load packages

```
library(tidyverse) # For data wrangling and ggplot
library(readxl)    # For reading Excel data
library(deSolve)   # For ODEs
```

Load and plot data

The data are available in the file *04_Joth_17_WS_data_for_R-script.xlsx*. I do the following:

- Compute the fractional conversion as Glucose/Maxglucose;
- Make a categorical version of *Dose* (mainly/only for plot purposes) and also a version with values 5/10/15:
- Make a plot of the data.

```r
dat1 <- read_excel("04_Joth_17_WS_data_for_R-script.xlsx")
dat1 <- dat1 %>% mutate(FracConv=Glucose/Maxglucose,
                        DoseGrp=factor(round(Dose,2)),
                        DoseGrp2=factor(rep(c(5,10,15),each=5))) # New variables
dat1 %>% head()
```

```
## # A tibble: 6 x 8
##    Dose  Time Glucose Maxglucose    E0 FracConv DoseGrp DoseGrp2
##   <dbl> <dbl>   <dbl>      <dbl> <dbl>    <dbl> <fct>   <fct>
## 1  1.76     0    0          31.2  49.9    0     1.76    5
## 2  1.76    24    5.08       31.2  49.9    0.163 1.76    5
## 3  1.76    48    7.08       31.2  49.9    0.227 1.76    5
## 4  1.76    72    8.91       31.2  49.9    0.286 1.76    5
## 5  1.76   144   11.0        31.2  49.9    0.353 1.76    5
## 6  3.53     0    0          31.2 100.     0     3.53    10
```

```r
dat1 %>% summary()
```

```
##       Dose            Time          Glucose         Maxglucose
##  Min.   :1.764   Min.   :  0.0   Min.   : 0.000   Min.   :31.18
##  1st Qu.:1.764   1st Qu.: 24.0   1st Qu.: 6.083   1st Qu.:31.18
##  Median :3.527   Median : 48.0   Median :11.018   Median :31.18
##  Mean   :3.527   Mean   : 57.6   Mean   :10.249   Mean   :31.18
##  3rd Qu.:5.291   3rd Qu.: 72.0   3rd Qu.:15.327   3rd Qu.:31.18
##  Max.   :5.291   Max.   :144.0   Max.   :21.504   Max.   :31.18
##        E0            FracConv      DoseGrp  DoseGrp2
##  Min.   : 49.90   Min.   :0.0000   1.76:5   5 :5
##  1st Qu.: 49.90   1st Qu.:0.1951   3.53:5   10:5
##  Median :100.08   Median :0.3533   5.29:5   15:5
##  Mean   : 99.98   Mean   :0.3287
##  3rd Qu.:149.97   3rd Qu.:0.4915
##  Max.   :149.97   Max.   :0.6896
```

```r
dat1 %>% ggplot(aes(x=Time, y=FracConv, color=DoseGrp2)) + geom_point() + geom_line() +
  ylab("Fractional conversion") + scale_color_manual(values=c("blue", "red", "green"))
```

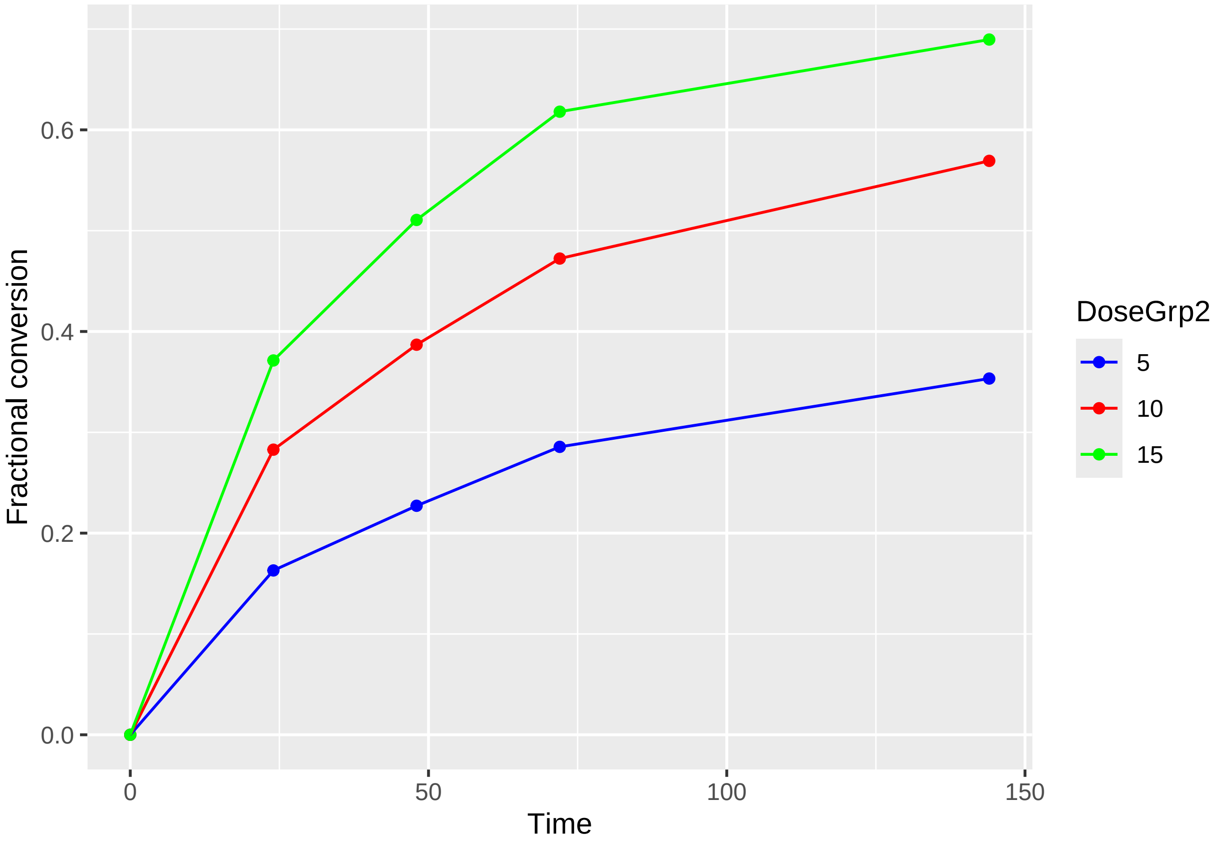

External/fixed parameters

I make a vector with the external/fixed parameters in the model. It turns out to be convenient to have these values saved as a vector (*KnownPar*) as well as as a data frame (*KnownParData*).

```
# External/fixed parameters
KnownPar <- c(KGa=13.0, KG2=3.302364458,  Ks=41.8243522, KGb=1, Km=0.2, kcat=100,
              X0=0, KX=76.09999118, I0=0, KI=1000, kIa=0, kib=0, kIb=0,
              C0=28.35, fBG=0.05, Gmax=0.990462478)
KnownPar
```

```
##          KGa          KG2           Ks          KGb           Km         kcat
##   13.0000000    3.3023645   41.8243522    1.0000000    0.2000000  100.0000000
##           X0           KX           I0           KI          kIa          kib
##    0.0000000   76.0999912    0.0000000 1000.0000000    0.0000000    0.0000000
##          kIb           C0          fBG         Gmax
##    0.0000000   28.3500000    0.0500000    0.9904625
```

```
# As dataframe (for convenience when certain values are extracted)
KnownParData <- KnownPar %>% t() %>% data.frame()
KnownParData
```

```
##   KGa      KG2       Ks KGb  Km kcat X0       KX I0   KI kIa kib kIb    C0  fBG
## 1  13 3.302364 41.82435   1 0.2  100  0 76.09999  0 1000   0   0   0 28.35 0.05
##        Gmax
## 1 0.9904625
```

ODE

Set-up I use the tools from the package called *deSolve*. In order to specify the ODE model, we need:

- The ODE model itself, including the derivatives and possibly also derived variables. Notice that the ODEs for Ea and BG are just linear ODEs;
- The initial conditions (states);
- Values of parameters entering into the ODEs. This includes the known parameters (above) as well as *kia* and *ks* (to be estimated).

```
# ODE model
odeModel <- function(t, state, parameters)
  {
  with(as.list(c(state, parameters)), {
    dS <- -ks*Ea*S / (S+Ks*(1+G/KGa+G2/KG2+X0/KX+I0/KI))
    dG2 <- -dS * 342 / 324 - kcat*BG*G2 / (G2+Km*(1+G/KGb))
    dG <- kcat * BG * G2 * 360 / 342 / (G2+Km*(1+G/KGb))
    dEa <- -(kia+kIa+I0) * Ea
    dBG <- -(kib+kIb+I0) * BG
    Conv <- (1-S/C0)*Gmax
    list(c(dS,dG2,dG,dEa,dBG),Conv)
  })
}

# Initial conditions for first dose
state1 <- c(S=KnownParData$C0,
            G2=0,
            G=0,
            Ea=dat1$E0[1]/1000,
            BG=KnownParData$fBG*dat1$E0[1]/1000)
state1
```

```
##          S         G2          G         Ea         BG
## 28.3500000  0.0000000  0.0000000  0.0498960  0.0024948
```

Example For illustration, I choose some values (kia=0.02 and ks=15, respectively), and solve the associated ODEs.

Notice:

- The last column is the fractional conversion (just denoted "6" here);
- One can use different numerical methods. Here, I try the so-called Domand-Price ("ode45") and Runge-Kutta of order 4 ("rk4"). They only differ quite far out in the decimals, but Domand-Price is must faster so I am going to use that one below;
- I only ask for the values at the observed time points, but values are computed at a fine time grid nonetheless (but not returned). Results are bad if only time points with observations are used.

```
# Domand-Price: Control with atol
ode(y = state1, times=c(26,48,72,144), func=odeModel, parms=c(KnownPar,kia=0.02,ks=15),
    method="ode45", atol=1e-6)
```

```
##   time        S        G2        G          Ea        BG         6
## 1   26 28.35000 0.0000000 0.000000 0.049896000 0.0024948 0.0000000
## 2   48 24.15586 1.1052954 3.496686 0.032134841 0.0024948 0.1465304
## 3   72 21.88673 0.8054947 6.333527 0.019884506 0.0024948 0.2258071
## 4  144 19.40662 0.1766340 9.751156 0.004711192 0.0024948 0.3124543
```

```
# Runge-Kutta of order 4: Control with hini
ode(y=state1, times=c(26,48,72,144), func=odeModel, parms=c(KnownPar,kia=0.02,ks=15),
    method="rk4", hini=0.01)
```

```
##   time        S        G2        G           Ea        BG         6
## 1   26 28.35000 0.0000000 0.000000 0.049896000 0.0024948 0.0000000
## 2   48 24.15586 1.1052984 3.496682 0.032134841 0.0024948 0.1465304
## 3   72 21.88673 0.8054954 6.333525 0.019884506 0.0024948 0.2258070
## 4  144 19.40662 0.1766334 9.751155 0.004711191 0.0024948 0.3124543
```

```
# Only uses time the given time points: Bad!
# ode(y=state1, times=c(26,48,72,144), func=odeModel,
#     parms=c(KnownPar,kia=0.02,ks=15), method="rk4")
```

If I ask for values at more time values, I can also plot the solutions and get smooth curves. For example:

```
ode(y=state1, times=1:145, func=odeModel, parms=c(KnownPar,kia=0.02,ks=15),
    method="ode45", atol=1e-6)  %>% plot()
```

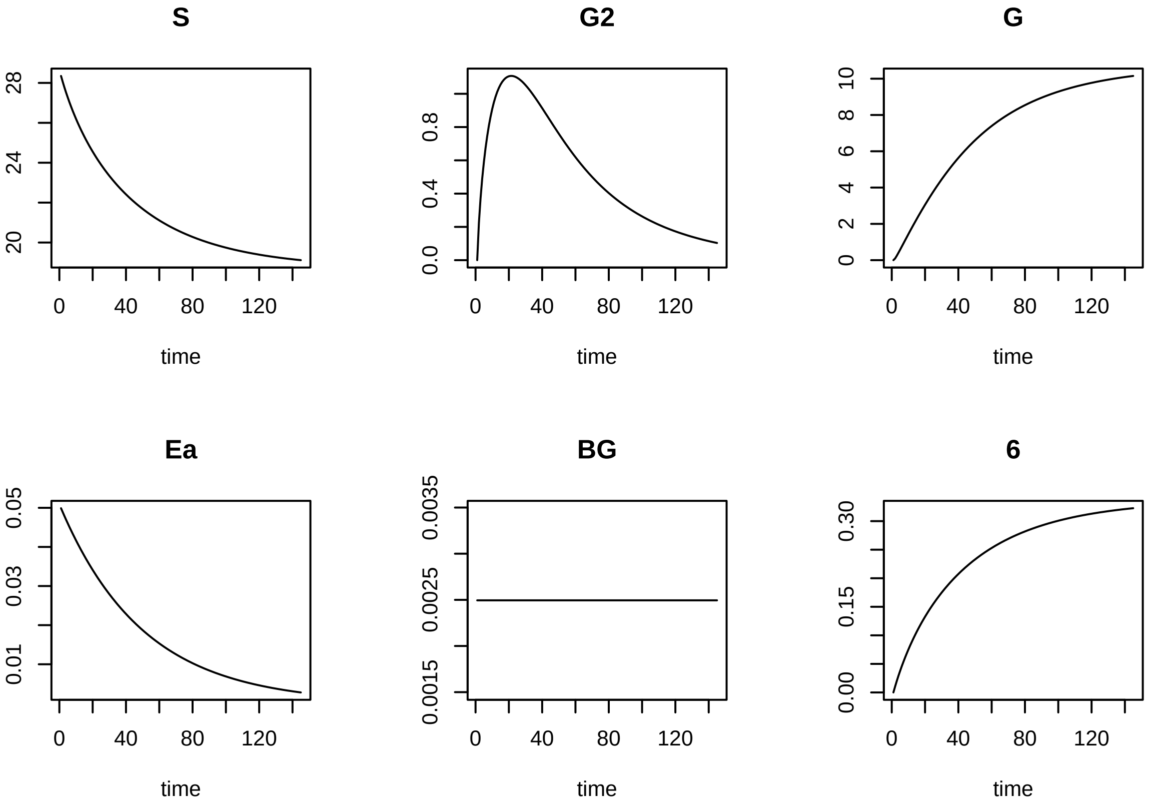

Non-linear regression

The next task is to incorporate the ODE solution into a non-linear regression.

The most obvious strategy would be to define a regression function and use that in the *nls* function. Unfortunately, the regression function does not play along with *nls*, so I make a manual implementation of the loss function (sum of squared residuals) and minimize it.

More specifically,

- I implement the least squares loss function, and optimize it to get estimates for *kia* and *ks*;

- I compute the residual sum of squares (RSS) and thereafter the estimated residual standard deviation (sigma);
- I compute standard errors and 95% confidence intervals for *kia* and *ks* (as is common, I do not compute SE and CI for sigma)
- I list the results in a data frame.

Notice:

- The dataset must be on the "long format", i.e., one line per measurement;
- The dataset must include variables called *Time*, *E0*, and *FracConv*;
- I leave out the observations from time t=0. This is because the fractional conversion is zero by construction (so no real measurement are taken). It does not change the estimates, but it changes the standard errors and confidence intervals;
- I need starting values for the minimization problem; they are given to the functions as arguments *kia* and *ks*. If the problem is well-behaved the solution should not depend on these starting values, and one can check different sets of starting values to check for robustness.

Here comes the function that does the work:

```
# Input:
# - data: dataset with variables Dose, Time, FracConv, E0, C0 (and possibly more)
# - knownpar: names vector with the preset/known parameters, namely
#             KGa,KG2,Ks,KGb,Km,kcat,X0,KX,I0,KI,kIa,kib,kIb,fBG,Gmax
# - kia0, ks0: Starting values for minimization
# Output: Data frame with estimates, SE, CI
EstFct <- function(data, knownpar, kia0, ks0)
{
  data <- data %>% filter(Time>0)         # Do not use zeroes from time zero
  n <- dim(data)[1]                       # No of observations
  knownpardata <- data.frame(t(knownpar)) # Known parameters as data frame

  # The loss function as function of par=(kia,ks)
  lossFct <- function(par)
  {
     kia <- as.numeric(par[1]) # Extract kia
     ks <- as.numeric(par[2])  # Extract ks

     fVals <- rep(NA,n) # Vector for the function values
     for (i in 1:n)     # Run through all observations
     {
      # Initial states
      state <- c(S=knownpardata$C0, G2=0, G=0, Ea=data$E0[i]/1000,
                 BG=knownpardata$fBG*data$E0[i]/1000)
      # Solve ODE
      out <- ode(y=state, times=c(0,data$Time[i]), func=odeModel,
                 parms=c(knownpar,kia=kia,ks=ks),
                 method="ode45", atol=1e-6)
      # Extract fractional conversion at relevant time
      fVals[i] <- data.frame(out)$X6[2]
     }
    # Use RSS as loss and return it
    loss <- sum((data$FracConv - fVals)^2)
    return(loss)
```

```
  }
  # Minimize RSS loss
  opt <- optim(c(0.01,10), fn=lossFct, lower=c(0,0), method="L-BFGS-B", hessian=TRUE)

  sigmaSqr <- opt$value/(n-2)                             # Residual variance
  StdError <- sqrt(sigmaSqr * diag(solve(opt$hessian))) # SEs via inverse Hessian

  # Prepare output as data frame
  res <- data.frame(Estimate=opt$par, StdError=StdError)
  res[3,] <- c(sqrt(sigmaSqr),NA)
  row.names(res) <- c("kia","ks","sigma")
  res <- res %>% mutate(Lower95=Estimate-1.96*StdError, Upper95=Estimate+1.96*StdError)
  return(res)
}
```

Estimation for our data

Finally, I use it for the data at hand:

```
# Takes about half a minute to run
estData <- EstFct(data=dat1, knownpar=KnownPar, kia0=10, ks0=10)
estData
```

```
##          Estimate     StdError    Lower95     Upper95
## kia    0.01550036 0.0008847755  0.0137662  0.01723452
## ks    17.73265578 0.5816412561 16.5926389 18.87267264
## sigma  0.01371212           NA         NA          NA
```

Plot of solution

I make a function that can be used for plotting. It could probably be simplified. . .

```
# Input:
# - data: Dataset
# - kia.est, ks.est, knownpar: Parameter values (estimates and fixed)
# - tVec: time values where regression function is computed
# Output: Graph
PlotFit3 <- function(data,kia.est,ks.est,knownpar,E01,E02,E03,E04,tVec=0:150)
{
  # tVec: Values where regression function is computed
  knownpardata <- data.frame(t(knownpar)) # Known parameters as data frame

  # First dose: Set initial condition, solve SDE, save as data frame, include time vector
  state1 <- c(S=knownpardata$C0, G2=0, G=0, Ea=E01/1000, BG=KnownParData$fBG*E01/1000)
  out1data <- ode(y=state1, times=tVec, func=odeModel,
                  parms=c(knownpar,kia=kia.est,ks=ks.est),
                  method="ode45",atol=1e-6) %>%
    data.frame()
  out1data$Time <- tVec

  # Same for second dose
  state2 <- c(S=knownpardata$C0, G2=0, G=0, Ea=E02/1000, BG=KnownParData$fBG*E02/1000)
  out2data <- ode(y=state2, times=tVec, func=odeModel,
                  parms=c(knownpar,kia=kia.est,ks=ks.est),
                  method="ode45",atol=1e-6) %>%
```

```
    data.frame()
  out2data$Time <- tVec

  # Same for third dose
  state3 <- c(S=knownpardata$C0, G2=0, G=0, Ea=E03/1000, BG=KnownParData$fBG*E03/1000)
  out3data <- ode(y=state3, times=tVec, func=odeModel,
                  parms=c(knownpar,kia=kia.est,ks=ks.est),
                  method="ode45",atol=1e-6) %>%
    data.frame()
  out3data$Time <- tVec

  # Plot data points and the four estimated regression curves
  dat1 %>%
    ggplot(aes(x=Time, y=FracConv, color=DoseGrp)) +
      geom_point() +
      ylab("Fractional conversion") +
      scale_color_manual(values=c("blue", "red", "green")) +
      geom_line(data=out1data, aes(x=Time, y=X6), color="blue") +
      geom_line(data=out2data, aes(x=Time, y=X6), color="red") +
      geom_line(data=out3data, aes(x=Time, y=X6), color="green") +
      theme(legend.position="none")
}
```

Application of the function to our data gives the following plot:

```
PlotFit3(data=dat1, kia.est=estData$Estimate[1], ks.est=estData$Estimate[2],
         knownpar=KnownPar, E01=dat1$E0[1], E02=dat1$E0[6], E03=dat1$E0[11])
```

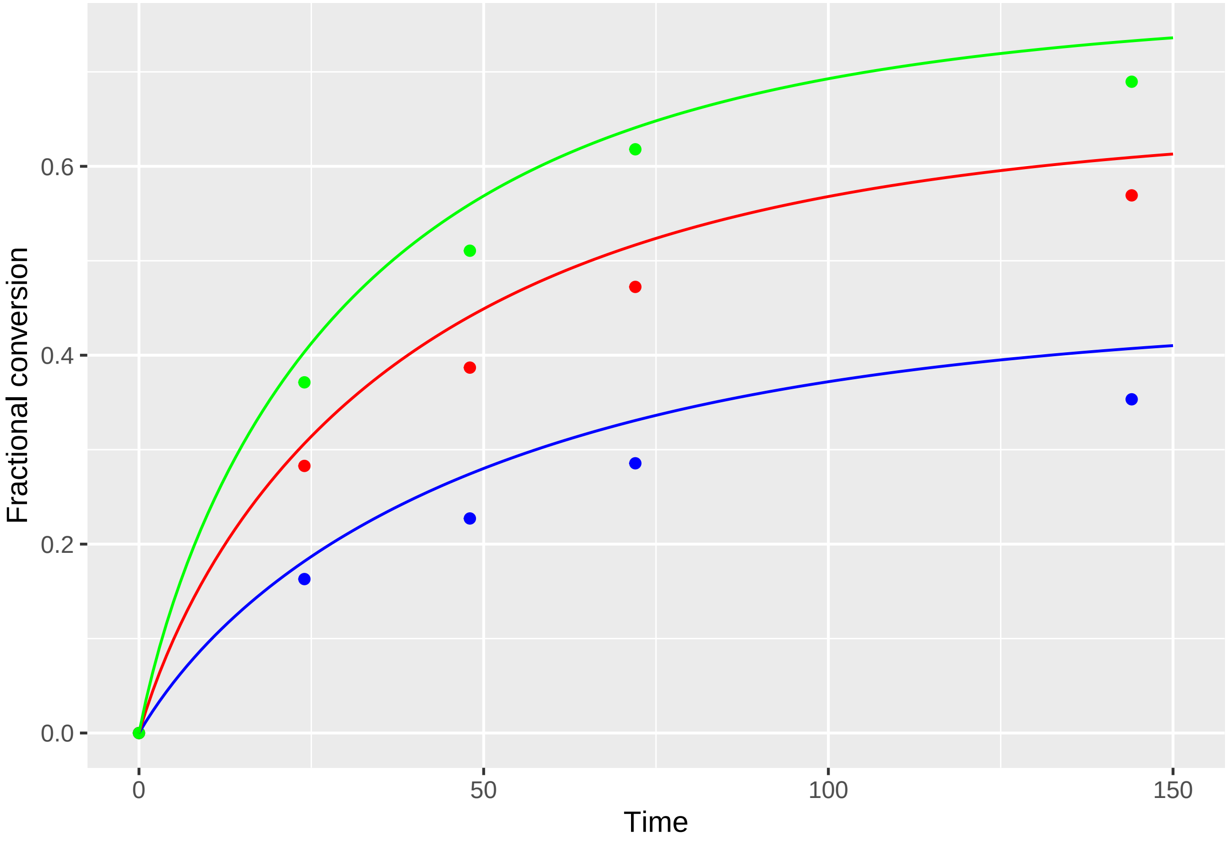

Supplement: S1 File — (PDF) [file pone.0312219.s007.pdf]
