# Supplementary material for: Enzymatic saccharification of peat polysaccharides is limited by accessibility
Source: PLoS One. 2025 May 23;20(5):e0312219. doi: 10.1371/journal.pone.0312219 (PMC12101845; doi:10.1371/journal.pone.0312219)
Supplement: S2 File — (PDF) [file pone.0312219.s008.pdf]

S2 File. Estimation of glucan fraction in soluble part of peat pretreated at 180 °C.

There was a difference of 19 mg glucan / g DM between the insoluble part (the washed substrate) and the unseparated peat pretreated at 180 °C (table 1). Calculation of the glucose concentration in the soluble part to glucan, before CTec3 treatment, gave 6 mg glucan / g DM. After CTec3 treatment, 20 mg glucan / g DM was calculated. Thus, there was 14 mg glucan / g DM in the soluble part, likely in the form of oligosaccharides. Pretreated peat therefore clearly seem to contain a soluble glucan fraction that made up 11% of the total glucan fraction. Also, the total amount of calculated glucan in the soluble part was 20 mg / g DM which explains the difference of 19 mg glucan difference between the insoluble and unseparated peat from the composition analysis.
